# A Short-Term High-Fat Diet Worsens Insulin Sensitivity with Changes in Metabolic Parameters in Non-Obese Japanese Men

**DOI:** 10.3390/jcm12124084

**Published:** 2023-06-16

**Authors:** Satoshi Kadowaki, Yoshifumi Tamura, Daisuke Sugimoto, Hideyoshi Kaga, Ruriko Suzuki, Yuki Someya, Nozomu Yamasaki, Motonori Sato, Saori Kakehi, Akio Kanazawa, Ryuzo Kawamori, Hirotaka Watada

**Affiliations:** 1 Department of Metabolism & Endocrinology, Juntendo University Graduate School of Medicine, 2-1-1 Hongo, Bunkyo-ku, Tokyo 113-8421, Japan; 2 Sportology Center, Juntendo University Graduate School of Medicine, 2-1-1 Hongo, Bunkyo-ku, Tokyo 113-8421, Japan; 3 Sports Medicine & Sportology, Juntendo University Graduate School of Medicine, 2-1-1 Hongo, Bunkyo-ku, Tokyo 113-8421, Japan

**Keywords:** insulin resistance, insulin clearance, non-obese, East Asian, gut microbiota, ectopic fat

## Abstract

A short-term high-calorie high-fat diet (HCHFD) impairs insulin sensitivity in non-obese South Asian but not Caucasian men; however, the effect of short-term HCHFD on insulin sensitivity in East Asians is unknown. We recruited 21 healthy non-obese Japanese men to evaluate metabolic parameters and gut microbiota before and after 6-day HCHFD consisting of a regular diet plus a 45% energy excess with dairy fat supplementation. We evaluated tissue-specific insulin sensitivity and metabolic clearance rate of insulin (MCRI) using a two-step hyperinsulinemic euglycemic clamp, glucose tolerance using the glucose tolerance test, and measured ectopic fat in muscle and the liver using ¹H-magnetic resonance spectroscopy. The primary outcome of this study was insulin sensitivity measured by the clamp study. The secondary/exploratory outcomes were other metabolic changes. After HCHFD, levels of circulating lipopolysaccharide binding protein (LBP), a marker of endotoxemia, increased by 14%. In addition, intramyocellular lipid levels in the tibialis anterior and soleus and intrahepatic lipid levels increased by 47%, 31%, and 200%, respectively. Insulin sensitivity decreased by 4% in muscle and 8% in liver. However, even with reduced insulin sensitivity, glucose metabolism was maintained by increased serum insulin concentrations due to lower MCRI and higher endogenous insulin secretion during the clamp. Glucose levels during the meal tolerance test were comparable before and after HCHFD. In conclusion, short-term HCHFD impaired insulin sensitivity in the muscle and livers of non-obese Japanese men with increased LBP and ectopic fat accumulation. Elevated insulin levels from modulated insulin secretion and clearance might contribute to the maintenance of normal glucose metabolism during the clamp and meal tolerance test.

## 1. Introduction

The number of people with type 2 diabetes has been increasing worldwide [1]. Insulin resistance due to obesity is a core pathology of metabolic syndrome. It is generally present before the onset of diabetes [2]. Asians have a higher propensity to develop diabetes. Several studies have suggested that insulin resistance is occasionally observed in non-obese Asians in whom it elicits metabolic abnormalities [3,4,5,6]. Insulin resistance in the skeletal muscle of non-obese Japanese individuals is associated with dyslipidemia and hypertension [4]. However, the etiology of insulin resistance in non-obese Asians has not been fully elucidated.

Several studies have reported that high intake of saturated fatty acids increases the risk of type 2 diabetes [7,8]. They cause insulin resistance in vitro and in animal models [9]. Interestingly, even short-term intake (5–6 days) of a high-calorie, high-fat diet (HCHFD) containing saturated fatty acids can induce significant metabolic changes. These changes can vary among different ethnicities and even among individuals within the same ethnicity. This short-term dietary intervention allows us to study the initial metabolic responses to a high-fat diet, providing insights into the early stages of diet-induced insulin resistance. Bakker et al. reported that 5-day HCHFD caused peripheral insulin resistance in South Asians but not in Caucasians [10]. However, Brøns et al. reported that 5-day HCHFD caused hepatic, but not peripheral insulin resistance in non-obese Caucasians [11]. Studies in Germany [12] and the United States [13] also showed no significant changes in peripheral insulin sensitivity after HCHFD of 5 or 7 days in non-obese individuals. These data suggest that the effect of short-term HCHFD on insulin sensitivity might differ by ethnicity or organ.

Studies examining the metabolic effects of short-term high-fat diets have been limited to South Asians; data on East Asians are not yet available. Previous studies have indicated that South Asians tend to exhibit greater insulin resistance compared to East Asians [14,15,16]. Furthermore, the correlation between body fat percentage and insulin sensitivity has been shown to be stronger in East Asians than in South Asians [15]. This suggests that East Asians might be more affected by higher levels of adiposity, while in South Asians, non-obesity-related insulin resistance could be more prominent [15]. The reasons for these disparities may be attributed to genetic factors, as well as environmental factors, such as diet, physical activity levels, and sociocultural aspects that can impact epigenetics [17]. From these, it is crucial to investigate the metabolic effects of short-term HCHFD in East Asian populations to better understand the underlying mechanisms and potential implications for targeted prevention and treatment strategies in combating insulin resistance across different ethnic groups.

Although 5-day HCHFD causes peripheral insulin resistance in South Asians [10], the metabolic changes associated with insulin resistance caused by short-term HCHFD in East Asians have not been fully assessed. Short-term HCHFD might induce changes in microbiota composition or colonic inflammation. Thus, it might affect insulin sensitivity [18,19,20]. In addition, short-term HCHFD might enhance intramyocellular lipid (IMCL) and intrahepatic lipid (IHL) accumulation, which are frequently observed in non-obese subjects and might play a role in inducing insulin resistance [21,22]. Furthermore, the lower metabolic clearance rate of insulin (MCRI) often observed with insulin resistance could be an important initial metabolic change that accelerates obesity and insulin resistance [21,22]. On the other hand, it has been suggested that adiponectin and fibroblast growth factor-21 (FGF-21) levels increase after HCHFD and inhibit lipid accumulation and insulin resistance in muscle and the liver to compensate for lower insulin resistance [23,24,25,26,27]. However, it remains unclear whether short-term HCHFD alters insulin sensitivity and how changes in the metabolic parameters, including those mentioned above, are affected by short-term HCHFD in non-obese East Asians. Thus, we evaluated the effect of short-term HCHFD on insulin sensitivity and associated metabolic responses in non-obese Japanese individuals.

## 2. Materials and Methods

### 2.1. Study Participants

Twenty-one healthy, non-diabetic, non-obese Japanese men aged between 21 and 29 years were recruited on a voluntary basis between 2017 and 2018 for this study through posters or recruitment from two outsourcing companies (CROèe Inc., Tokyo, Japan, and Souken, Tokyo, Japan). Study eligibility was assessed with a detailed screening questionnaire that included medical history. The following exclusion criteria were applied: history of diabetes, history of food allergy, history of drug hypersensitivity, habitual drinker (ethanol intake ≥30 g/day), history of hypertension or dyslipidemia, history of gastrointestinal disease, history of gastrointestinal surgery, history of hepatitis B virus or hepatitis C virus infection, bleeding tendency, history of heart disease, liver or renal dysfunction, recent weight change or attempts to lose weight (3 kg change within the past month), low-carbohydrate diet (carbohydrate intake <150 g/day), and current smoker. The study protocol was approved by the ethics committee of the Faculty of Medicine of Juntendo University (approval number, 2016131; approval date, 5 October 2016) and conducted in accordance with the Declaration of Helsinki.

This study was registered in the UMIN Clinical Trials Registry as UMIN000029209. Written informed consent was obtained from all participants.

### 2.2. Study Design and Methods

**Figure 1:**
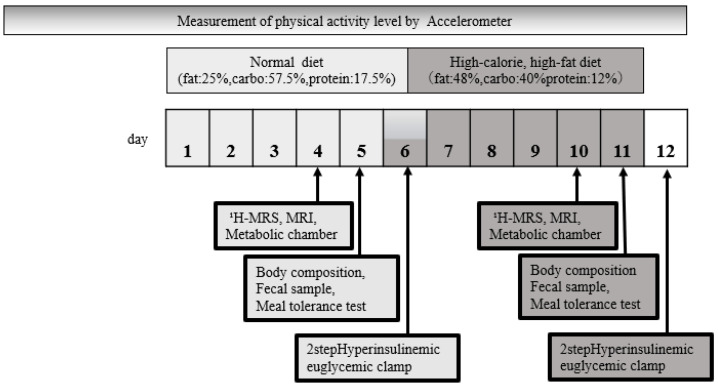
Study protocol. ^1^H-MRS: proton magnetic resonance spectroscopy, MRI: magnetic resonance imaging.

Figure 1 summarizes the study design of this single-arm, prospective exploratory intervention study. In our study, we established the post-normal diet condition as the baseline, with the primary objective being to assess subsequent changes following the HCHFD. Consequently, the normal diet and HCHFD were implemented in a continuous sequence. The study duration was 12 days. During days 1–5, participants were instructed to consume the standard diet delivered from a food company (Tokatsu Foods, Tokyo, Japan). The macronutrient composition of the standard diet consisted of approximately 25% energy from fat, 57.5% from carbohydrates, and 17.5% from protein. On day 4, subjects were instructed to visit Juntendo University at 14:00. We evaluated ectopic fat content in muscle and the liver using ¹H-magnetic resonance spectroscopy (MRS) and fat distribution using magnetic resonance imaging (MRI). Next, subjects entered the metabolic chamber at 16:00 and remained until 8:00 the following morning (day 5). Body composition was evaluated using dual-energy X-ray absorptiometry in the fasting state [28]. A fecal sample was collected. The meal tolerance test (0–240 min) was performed at 8 AM. On day 6, we performed the hyperinsulinemic euglycemic clamp test after an overnight fast [21,22]. Afterwards, subjects consumed HCHFD on days 6–11. During this period, subjects were instructed to eat cream (+45% extra energy) in addition to the standard diet. We used the same evaluation protocol on days 10–12 as on days 4–6 to evaluate the effects of HCHFD (Figure 1). The primary outcome of this study was insulin sensitivity measured by the hyperinsulinemic euglycemic clamp study. The secondary/exploratory outcomes were other metabolic changes. In determining the sample size for our study, we considered previous research and practical considerations, as this is the first study of its kind among East Asians. We aimed to recruit a similar number of participants as in previous HCHFD studies (12 to 26 subjects) [10,11,12,13], which had found several significant differences in insulin sensitivity. Consequently, we enrolled 21 subjects, a number consistent with the range reported in prior research.

### 2.3. Dietary and Physical Activity Manipulations

We measured mean physical activity levels using an accelerometer (Lifecorder; Suzuken, Nagoya, Japan). The accelerometer was provided to each subject on day 14. Mean daily physical activity level was evaluated over 7 days. Next, each subject was asked to maintain their daily physical activity level at the mean level ±10% during the 3 days before the experiment to reduce the effects of physical activity level on physiological measurements. The total energy requirement was estimated based on physical activity level and body composition [4]. It reflected the total energy intake of the standard diet. For HCHFD, we used cream to increase total energy intake by 45%. Thus, a surplus of approximately 1000 kcal/day was provided (~320 g/day), which consisted of approximately 70% saturated fatty acids (e.g., palmitic acid) and approximately 30% monounsaturated fatty acids. The HCHFD macronutrient composition was 48% of energy from fat, 40% from carbohydrates, and 12% from protein. To ensure compliance, subjects were instructed to take pictures of the food after each meal and send them by e-mail to investigators. Subjects were asked to refrain from alcohol during the study.

### 2.4. ^1^H-MRS and MRI

IHL levels in segment 6 of the liver and IMCL values of the right soleus (SOL) and tibialis anterior (TA) measures were measured using ^1^H-MRS [29,30]. IMCL was quantified based on methylene signal intensity (S-fat) using the creatine signal as the reference and calculated as the S-fat/creatine signal ratio. IHL was quantified based on S-fat, with water as the internal reference. IHL was calculated as follows: percentage of H_2_O + S-fat [S-fat × 100/(H_2_O + S-fat)] [29,30]. Visceral fat area and subcutaneous fat area were measured with MRI, as described previously [30]. Briefly, T1-weighted transaxial images were obtained. Visceral fat area and subcutaneous fat area in the fourth and fifth lumbar interspaces were measured as described previously using specific software (AZE Virtual Place Version 3.6, AZE Co., Tokyo, Japan) Version 3.6 [30].

### 2.5. Two-Step Hyperinsulinemic Euglycemic Clamp Procedure

After an overnight fast, a two-step hyperinsulinemic euglycemic glucose clamp study was performed with an artificial endocrine pancreas (STG 55 Nikkiso, Shizuoka, Japan). Briefly, after securing an intravenous cannula in the forearm, primed [6,6-^2^H_2_] glucose (200 mg/m^2^ body surface area (BSA), Cambridge Isotope Laboratories, Tewksbury, MA, USA) was given intravenously, followed by a constant infusion of 2 mg/m^2^ BSA per minute for 3 h (−180 to 0 min) to measure fasting endogenous glucose production (EGP) [31]. This was followed by a primed insulin infusion (40 mU/m^2^ per minute followed by 20 mU/m^2^ per minute; each for 5 min) and continuous insulin infusion at 10 mU/m^2^ per min for 3 h (first step) (0 to 180 min) [32,33]. During the second step, after administering a priming insulin infusion (80 mU/m^2^ per minute followed by 40 mU/m^2^ per minute; each for 5 min), we continuously infused insulin at 20 mU/m^2^ per minute for 3 h (180 to 360 min) [34,35]. The infusion of [6,6-^2^H_2_] glucose was decreased by 75% of the initial infusion rate during the first step and 85% of the basal rate during the second step of the clamp to maintain constant plasma glucose enrichment [34]. The plasma glucose levels in arterialized blood were maintained at 95 mg/dL by varying infusion rates of 20% glucose containing 2.5% [6,6-^2^H_2_] glucose. The artificial endocrine pancreas could measure blood glucose levels continuously. Blood samples were collected for biochemical analysis at 10 min intervals during the last 30 min before the clamp and steady-state periods of the first and second steps. The enrichment of [6,6-^2^H_2_] glucose in plasma was measured using high-performance liquid chromatography with a LTQ-XL-Orbitrap mass spectrometer (Thermo Fisher Scientific, Waltham, MA, USA) as described previously [36].

### 2.6. Determination of Tissue-Specific Insulin Sensitivity and MCRI with the Clamp Test

The steady-state equation was used to calculate the rate of EGP and the rate of disappearance of glucose (Rd) at each step [37]. EGP and Rd were normalized by BSA [35] and fat-free mass [32], respectively. Because EGP is suppressed in accordance with insulin concentration at low insulin levels (~20 mU/mL) [38], we divided the percentage of reduction in EGP in the first step by the steady-state serum insulin (SS_SI_) level during the glucose clamp. We used the result as an index of hepatic insulin sensitivity [39]. Rd is enhanced in parallel with serum insulin concentrations [38]; therefore, Rd during the second step was divided by SS_SI._ This ratio was used as an index of muscle insulin sensitivity [40]. Adipose tissue insulin resistance (Adipo-IR) was calculated as fasting insulin × fasting free fatty acid (FFA) concentration. The metabolic clearance rate for serum insulin (MCRI) during the glucose clamp of the second step was calculated using the following equation [41]: MCRI = (IIR/[SS_SI_ 2 (B_SI_ × SS_SC_/B_SC_)]), where IIR is the insulin infusion rate, SS_SI_ is the steady-state serum insulin during the glucose clamp, B_SI_ is the basal serum insulin concentration, SS_SC_ is the steady-state serum C-peptide (CPR) concentration during the glucose clamp, and B_SC_ is the basal serum CPR concentration.

### 2.7. Biochemical Tests

Non-esterified fatty acids (Sekisui Medical Co., Ltd., Tokyo, Japan) and 3-hydroxybutyric acid (Kainos Laboratories, Inc., Tokyo, Japan) were measured using enzymatic methods. Plasma insulin, CPR, and interleukin (IL)-6 levels were measured with chemiluminescent enzyme immunoassays (Fujirebio Inc., Tokyo, Japan). Plasma glucagon (Mercodia AB, Uppsala, Sweden), glucagon-like peptide (GLP)-1(Merck Millipore, Tokyo, Japan), and gastric inhibitory polypeptide (GIP) (Immuno-Biological Laboratories Co., Ltd., Gunma, Japan) levels were measured using enzyme-linked immunosorbent assays (ELISAs). Serum lipopolysaccharide binding protein (LBP) (COSMO BIO Co., Ltd., Tokyo, Japan), adiponectin, tumor necrosis factor-α, monocyte chemoattractant protein (MCP)-1 (Funakoshi Co., Ltd., Tokyo, Japan), and FGF-21 (Bio-Techne, Tokyo, Japan) levels were measured using ELISAs.

### 2.8. Fecal Sample Evaluation

The fecal samples were dissolved in a preservation solution containing guanidine thiocyanate (Feces Collection kit; Techno Suruga Lab, Shizuoka, Japan). The samples were separated into 10 aliquots (100–200 mg) in screw cap tubes and immediately stored at −80 °C. These fecal samples were sent to Takara Bio for genome extraction (NucleoSpin Microbial DNA, Takara Bio, Shiga, Japan) and 16S rRNA analysis.

### 2.9. 16S Metagenomic Sequencing

Two-step PCR was performed on purified DNA samples to obtain sequence libraries. The first PCR was performed for amplification using the 16S (V3–V4) Metagenomic Library Construction Kit for NGS (Takara Bio, Kusatsu, Japan) with these primer pairs: 341F(5′-TCGTCGGCAGCGTCAGATGTGTATAAGAGACAGCCTACGGGNGGCWGCAG3′) and 806R(5′-GTCTCGTGGGCTCGGAGATGTGTATAAGAGACAGGGACTACHVGGGTWTCTAAT-3′). They correspond to the V3–V4 region of the 16S rRNA gene. The second PCR was performed to add index sequences for the Illumina sequencer with a barcode sequence using the Nextera XT Index kit (Illumina, San Diego, CA, USA). The libraries were subjected to sequencing of 250 paired-end bases using the Miseq Reagent v3 kit and the Miseq (Illumina) at the Biomedical Center at Takara Bio as previously described [42]. The α-diversity, which includes observed species, Chao1, and Shannon phylogenetic diversity indices, was assessed and analyzed utilizing the Wilcoxon rank sum test. The β-diversity was approximated through the application of the weighted UniFrac metric, a method that measures the distance between different samples [43].

### 2.10. Whole-Body Indirect Calorimetry

Energy metabolism and respiratory quotient (RQ) before and after HCHFD were measured with an indirect calorimeter (metabolic chamber). Whole-body indirect calorimetry with an improved transient response was performed as described in previous studies [44,45]. The dimensions of the airtight chamber for the whole-body indirect calorimeter were 3.60 m width × 2.50 m depth × 2.65 m height with an internal volume of 21.30 m^3^ (FHC-15S, Fuji Medical Science, Kashiwa, Japan). The measurement of O_2_ and CO_2_ in the outgoing air was performed with online process mass spectrometry (VG Prima δB, Thermo Electron, MA, USA) was performed as described previously [46]. Every minute, the O_2_ consumption (VO_2_) and CO_2_ production (VCO_2_) rates were calculated using an algorithm for the improved transient response. The energy expenditure and RQ were calculated from VO_2_ and VCO_2_ as described previously [47,48]. The ambient temperature inside the metabolic chamber was set to 25 °C. The humidity was set to 50% as described previously in detail [49]. Each subject stayed in a room-sized respiratory chamber for 15 h. All participants entered the metabolic chamber at 16:00 on the first day of the measurement and sat in a resting position from 16:15 to 16:45 (4 h after lunch) to measure resting metabolism. All subjects had dinner at 17:00 and continued sitting in the room. All subjects were instructed to record details of their movement such as the time they went to the bathroom. The lights were turned off at 23:00. All subjects went to bed and woke up at 6 AM on the next morning. The subjects were instructed to remain resting on the bed from 6:15 to 7:00 to measure their basal metabolic rate. All subjects left the room at 7:30. During their time in the metabolic chamber, subjects were instructed to remain as inactive as possible by sitting on a chair. Energy consumption and RQ were calculated using the Harris–Benedict equation. Sleep RQ was evaluated as the mean value during the 3 h period from approximately 3:00 AM, which was considered to be associated with the lowest nighttime energy consumption.

### 2.11. Meal Tolerance Test

All subjects underwent a meal tolerance test. On days 5 and 12, test meals with the same calorie count and composition were provided. The total energy was 737 kcal. The meal had the following macronutrients: protein, 35.3 g; fat, 19.5 g; and carbohydrates, 104.4 g. The calorie count was set as a reference based on the Japanese standard dietary intake. Insulin, CPR, glucose, and intestinal hormone levels were measured from 0 to 240 min. Using meal tolerance test data, the insulin sensitivity index (ISI) comp was calculated as an index of insulin sensitivity [50]. In addition, insulinogenic index and area under the curve (AUC)-insulin/AUC-glucose were calculated and used as indices of early-phase insulin secretion and β-cell function, respectively [51,52]. We calculated the total AUC using the trapezoidal method.

### 2.12. Statistical Analysis

Data are presented as means ± SD. The Wilcoxon signed-rank test was used to assess mean differences before and after the HCHFD for all parameters and at each time point during the meal load test. Correlation analyses were performed using Spearman correlation coefficients. All statistical tests were two-sided, with a 5% significance level. We used SPSS Statistics for Windows, version 25.0. (IBM Corp., Armonk, NY, USA) for statistical analyses.

## 3. Results

### 3.1. Anthropometric and Metabolic Characteristics before and after Short-Term HCHFD

The anthropometric and metabolic characteristics of the 21 non-obese healthy male study participants (mean age, 23.8 ± 3.1 years) at baseline and after 6 days of HCHFD are shown in Table 1. None of the subjects reported recent illness or use of antibiotics. Body weight was slightly but significantly increased after HCHFD, primarily due to increased fat-free mass. Their fasting serum glucose, total cholesterol, high-density lipoprotein cholesterol, and total bile acid levels were significantly higher, while triglyceride levels were unchanged. The fasting levels of FFA, total ketone bodies, acetoacetic acid, 3-hydroxyacetic acid and γ-glutamyl trans peptidase were significantly lower after HCHFD. The total and high molecular weight adiponectin, leptin, and FGF-21 levels were significantly increased after HCHFD.

Circulating LBP, a marker of endotoxemia, increased significantly by 14% after HCHFD. High-sensitivity C-reactive protein (CRP) levels tended to be higher. The levels of tumor necrosis factor-α, IL-6, and MCP-1 were unchanged. In addition, the percent change in high-sensitivity CRP and percent change in LBP were significantly correlated (rs = 0.529, *p* = 0.014).

### 3.2. Change in Fat Distribution after Short-Term HCHFD

As shown in Table 2, IMCL in TA and SOL were significantly increased by 47 and 31%. IHL increased by 200%. There were no significant changes in abdominal visceral fat area and subcutaneous fat area.

### 3.3. Insulin Sensitivity Evaluated with a Two-Step Hyperinsulinemic Euglycemic Clamp at Baseline and after Short-Term HCHFD

**Figure 2:**
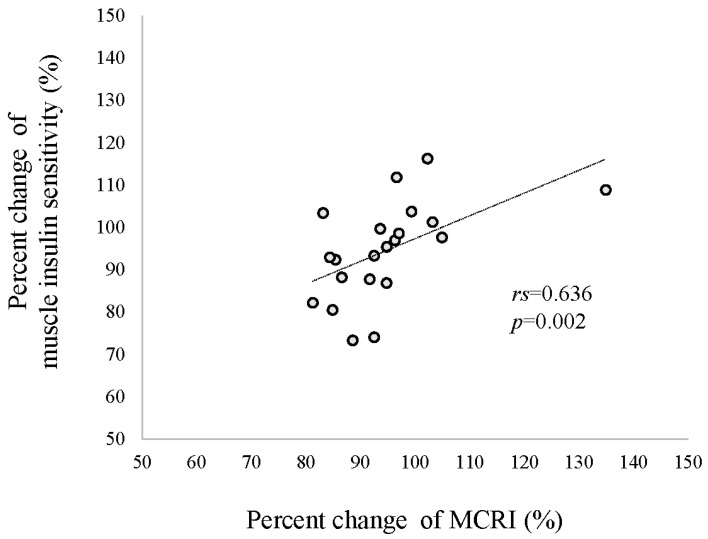
Relationship between percent change in muscle insulin sensitivity and metabolic clearance rate of insulin (MCRI). Correlation coefficients and *p* values are based on Spearman rank coefficients.

As shown in Table 2, SS_SI_ and SS_SC_ during the first and second steps were significantly increased after HCHFD. Consistently with increased SS_SI_, the MCRI was significantly decreased. The basal EGP increased significantly after HCHFD. The percent reduction in EGP during the first step was not significantly changed after HCHFD. However, hepatic insulin sensitivity, defined as percent reduction in EGP/SS_SI_ during the first step, was significantly lower after HCHFD. Similarly, Rd during the second step was unchanged after HCHFD. However, muscle insulin sensitivity, defined as Rd/SS_SI_ during the second step, was significantly lower. A significant correlation was observed between percent change of MCRI and muscle insulin sensitivity (rs = 0.636, *p* = 0.002) (Figure 2), while no significant correlation was found with hepatic insulin sensitivity (rs = 0.23, *p* = 0.925). Adipo-IR decreased after HCHFD.

These results indicate that HCHFD impairs insulin sensitivity in muscle and the liver. However, reductions in EGP during the first step and Rd during the second step were not altered by elevated SS_SI_ due to decreased MCRI and increased endogenous insulin secretion.

### 3.4. Meal Tolerance Test

**Figure 3:**
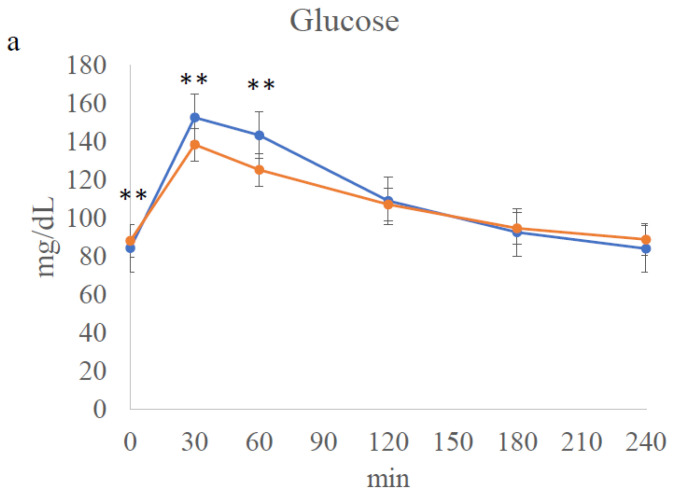

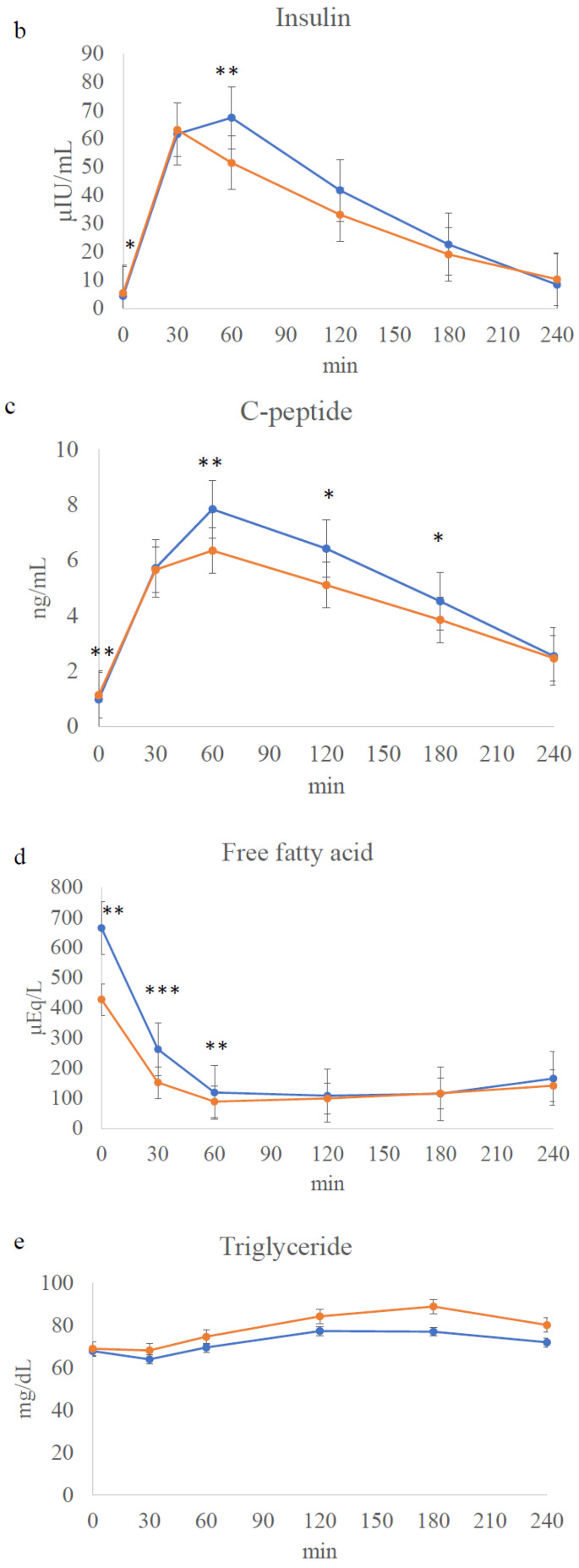

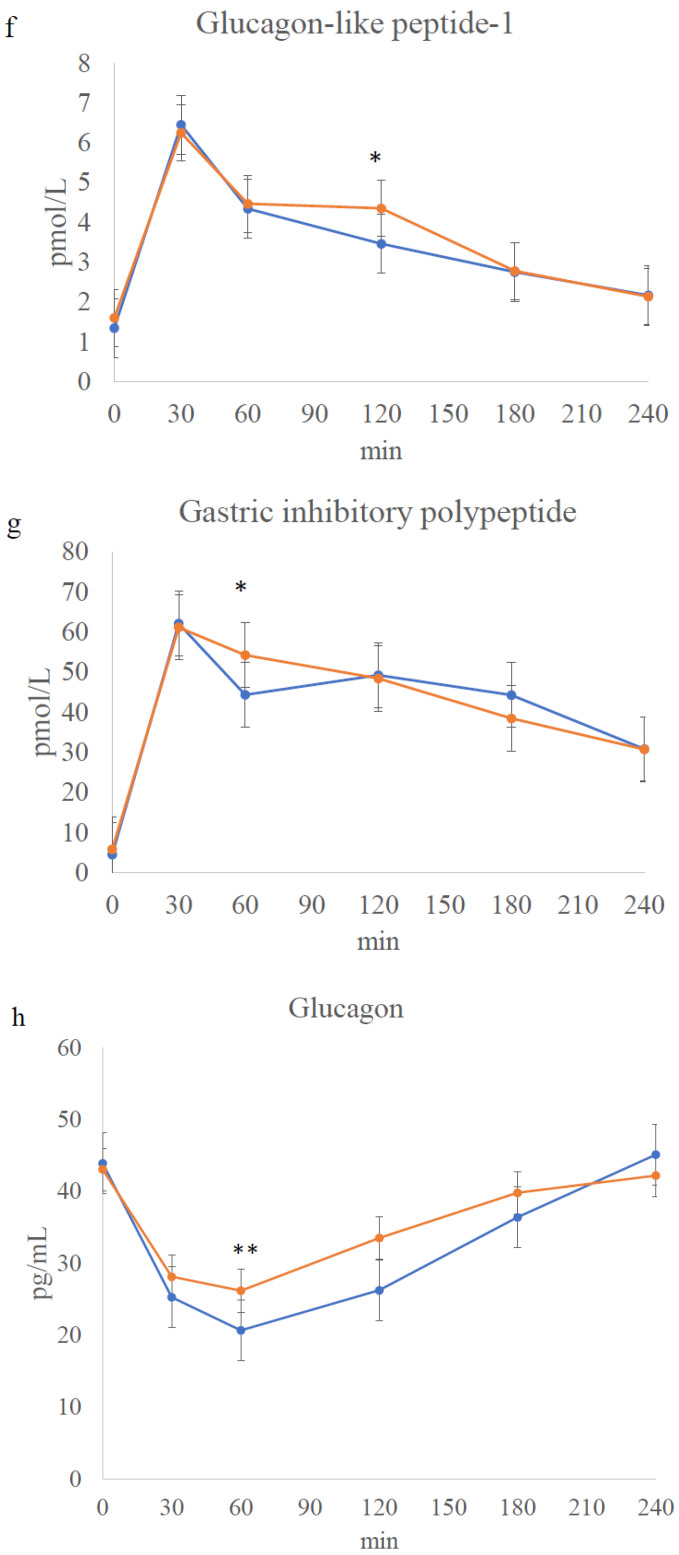
Metabolic parameters during the meal tolerance test. Each figure (**a**–**h**) represents the change during the test of the parameters indicated. Blue line: before high-calorie high-fat diet (HCHFD). Orange line: after HCHFD. *: *p* < 0.05. **: *p* < 0.01, ***: *p* < 0.001. (**a**); Glucose, (**b**); Insulin, (**c**); C-peptide, (**d**); Free fatty acid, (**e**); Triglyceride, (**f**); Glucagon-like Peptide-1, (**g**); Gastric Inhibitory Polypeptide, (**h**); Glucagon.

Figure 3 and Table 3 show metabolic parameters during the meal test. As shown in Figure 3, the fasting levels of glucose (84.4 ± 4.6 to 88.3 ± 6.1 mg/dL *p =* 0.005), insulin (4.4 ± 2.0 to 5.4 ± 2.5 μIU/mL *p =* 0.040), and CPR (1.0 ± 0.2 to 1.1 ± 0.3 ng/mL *p =* 0.006) were significantly higher (Supplementary Figure S1), and FFA levels (665.7 ± 174.9 to 428.3 ± 229.8 μEq/L *p* = 0.001) were lower after HCHFD. The insulinogenic index, calculated with both insulin and CPR, was significantly higher (Table 3), and plasma glucose levels at 30 and 60 min were significantly lower after HCHFD (Figure 3). Accordingly, serum insulin levels at 60 min, CPR levels at 60 and 120 min, AUC-insulin, and AUC-C-peptide were significantly decreased (Figure 3). As a result, AUC-glucose levels did not change significantly (Table 3). AUC-GIP, AUC-GLP-1, and AUC-glucagon did not change significantly after HCHFD (Table 3), while the levels of GIP at 60 min, GLP-1 at 120 min, and glucagon at 60 min were significantly higher after HCHFD (Figure 3). The FFA levels at 30, 60, and 240 min were significantly lower after HCHFD (Figure 3). AUC-FFA was also significantly lower after HCHFD (Table 3). Of note, the insulin sensitivity index (ISI comp) was unchanged after HCHFD (Table 3).

### 3.5. Effects of HCHFD on Gut Microbiota

**Figure 4:**
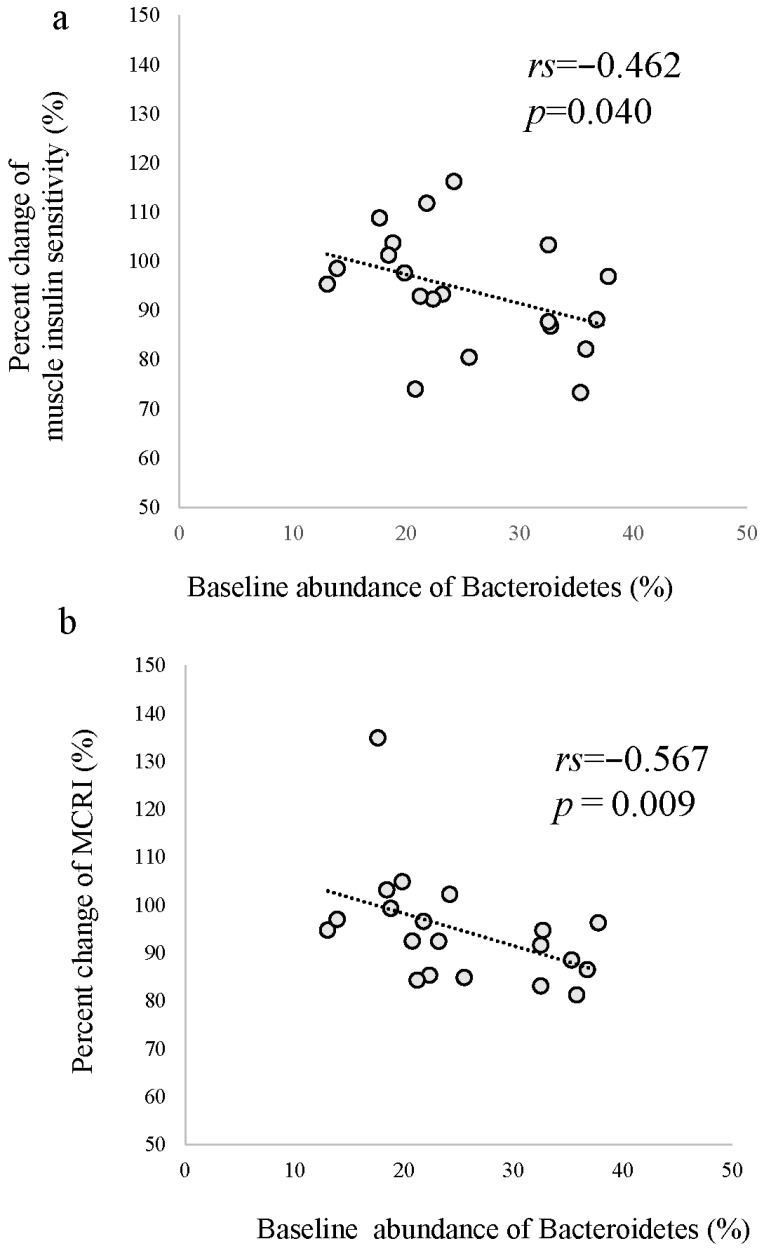
Correlation between baseline abundance of *Bacteroidetes* and percent change in muscle insulin sensitivity (**a**) and metabolic clearance rate of insulin (MCRI) (**b**). Correlation coefficients and *p* values were determined using Spearman rank coefficients.

Table 4 shows the abundance of gut microbiota at the phylum level at baseline and after short-term HCHFD. After HCHFD, the abundance of the phylum *Actinobacteria* was significantly higher. However, the change in abundance of *Actinobacteria* was not correlated with any metabolic changes. In addition, α and β diversity were not significantly changed after the HCHFD (Supplementary Figure S2). The baseline relative abundance of the phylum *Bacteroidetes*, which includes lipopolysaccharide (LPS)-producing Gram-negative gut microbiota, was correlated with changes in muscle insulin sensitivity (r = −0.46, *p* = 0.04) and MCRI (r = −0.57, *p* = 0.009) (Figure 4), while it was not significantly correlated with changes in LBP (r = −0.275, *p* = 0.24). The abundance of other phyla was not correlated with these metabolic parameters.

### 3.6. Energy Metabolism Evaluated Using the Metabolic Chamber at Baseline and after HCHFD

As shown in Table 1, the basal metabolic rate and RQ were unchanged after HCHFD. Although sleep energy expenditure was not altered, sleep RQ was significantly higher after HCHFD. In addition, percent change in sleep RQ was significantly correlated with percent change in fasting FFA (Supplementary Figure S3).

## 4. Discussion

In this study, we investigated the effect of HCHFD for 6 days on several metabolic parameters in healthy non-obese Japanese men. We found that this intervention resulted in a 14% increase in circulating LBP, a marker of endotoxemia; a 0.7% increase in body weight; 47% and 31% increases in IMCL in TA and SOL, respectively; and a 200% increase in IHL. The hyperinsulinemic euglycemic clamp test showed that liver and muscle insulin sensitivity decreased by 8% and 4%, respectively. However, a decrease in insulin clearance (MCRI) and an increase in endogenous insulin secretion (SS_SC_) were observed during the hyperinsulinemic euglycemic clamp test. Thus, the reduction in EGP during the first step and in Rd during second step of the hyperinsulinemic euglycemic clamp test were not affected by short-term HCHFD. Indeed, AUC-glucose levels during the meal tolerance test were comparable between baseline and after short-term HCHFD. Our data clearly demonstrate that short-term HCHFD induces insulin resistance in muscle and the liver in healthy non-obese Japanese men with several changes in metabolic parameters that suggest the propensity of East Asians to develop type 2 diabetes with a HCHFD.

The accumulation of IMCL and IHL is frequently observed in non-obese individuals with insulin resistance [21,22]. These accumulations are closely linked to the features of metabolic syndrome, even in non-obese subjects [33,53]. Several studies have demonstrated that short-term HCHFD increases IMCL [13] and IHL [10]; however, insulin sensitivities in liver and peripheral tissues determined with a glucose clamp were not altered, except in South Asians [10,11,13], and the increase in IMCL levels was relatively small (approximately 20%) [13]. The etiology of ectopic fat accumulation and insulin resistance in non-obese Asians remains unclear. Only one study has investigated the effect of short-term (5-day) HCHFD in non-obese South Asians. It showed that HCHFD causes IHL accumulation and peripheral insulin resistance, but not hepatic insulin resistance. In that study, IMCL levels were not measured. Thus, our study is the first to demonstrate that short-term HCHFD elicits ectopic fat accumulation and impaired insulin sensitivity in non-obese Asians. This difference in peripheral insulin sensitivity after HCHFD between Asians and Caucasians might be the reason why non-obese Asians readily develop metabolic diseases.

Previous studies have demonstrated that a high-fat diet induces intestinal inflammation, enhanced intestinal permeability, and endotoxemia that induces systemic insulin resistance [18,54]. Indeed, Kawano et al. demonstrated that mice with deficient expression of CCL2, a chemokine in intestinal cells, are protected from the deterioration in insulin sensitivity resulting from a high-fat diet [19]. A previous study demonstrated that short-term overfeeding with dairy cream does not modify gut permeability, fecal microbiota, plasma LBP and CRP levels, or peripheral insulin sensitivity in young healthy German men [12]. On the other hand, in this study, short-term HCHFD induced a significant increase in levels of circulating LBP, a marker of endotoxemia, and a non-significant increase in levels of CRP. In addition, the percent change in high-sensitivity CRP was correlated with that of LBP. Although a significant association between change in LBP levels and insulin sensitivity was not observed, the baseline relative abundance of *Bacteroidetes*, a type of LPS-producing gram-negative gut microbiota, was correlated with changes in both muscle insulin sensitivity and MCRI after HCHFD. Accordingly, LPS is induced by Gram-negative gut microbiota, which can cause endotoxemia [20]. These data suggest that intestinal inflammation induced by HCHFD might partly modulate insulin sensitivity and insulin clearance after HCHFD in healthy East Asian men, but not in healthy Caucasian men. This difference might explain the difference in the propensity to develop metabolic syndrome between Asians and Caucasians.

While our study provides valuable insights into the relationship between gut microbiota composition and metabolic changes induced by a short-term HCHFD in non-obese Japanese men, we recognize the limitations associated with focusing primarily on the phylum level. For instance, *Actinobacteria* play crucial roles in maintaining gut homeostasis; however, it is important to note that not all species within this phylum are beneficial. Some species can be opportunistic and potentially harmful, particularly when the gut ecosystem undergoes abnormal changes due to factors such as antibiotic usage, illness, and poor dietary habits [55]. As a result, our conclusions based on *Bacteroidetes* abundance should be interpreted with caution. Future studies employing a more comprehensive taxonomic resolution and advanced sequencing techniques may help elucidate the specific roles of individual bacterial taxa and their interactions in modulating insulin sensitivity and glucose metabolism, thereby deepening our understanding of the complex relationships between gut microbiota and host metabolism.

In the present study, decreases in liver and muscle insulin sensitivity were completely compensated by increased serum insulin concentration (SS_SI_) due to a decrease in insulin clearance (MCRI) and an increase in endogenous insulin secretion (SS_SC_) during hyperinsulinemia. Our previous cross-sectional study revealed that increased insulin levels due to decreased MCRI and enhanced endogenous insulin secretion completely compensate for moderate peripheral insulin resistance in healthy non-obese subjects [41,56]. An animal model showed that impairment of MCRI induces hyperinsulinemia. Such mice subsequently develop obesity and insulin resistance [57]. In addition, mice with genetic deletion of carcinoembryonic antigen-related adhesion molecules-1, a main regulator of MCRI, have hyperinsulinemia, obesity, and insulin resistance [58]. Thus, decreased MCRI could be an important initial metabolic change that accelerates subsequent obesity and insulin resistance. Therefore, in healthy Asian individuals, reduction in MCRI could be a primary change that induces insulin resistance rather than a change to compensate for insulin resistance. Indeed, Bakker et al. reported that MCRI was increased after 5 days of HCHFD in South Asians, but unchanged in Caucasians [10]. This difference might also explain the difference in the propensity to develop metabolic syndrome between Asians and Caucasians.

We observed a decrease in insulin sensitivity and MCRI during the clamp test after HCHFD. In contrast, during the meal tolerance test, both AUC-insulin and AUC-CPR were lower compared to baseline, with AUC-glucose remaining largely unchanged, although glucose levels were slightly decreased at several points during the meal test. A possible explanation for this discrepancy could be an increase in early-phase insulin secretion (e.g., insulinogenic index; Table 3) following the HCHFD, leading to a reduction in postprandial blood glucose levels. Consequently, the decreased glucose levels would result in lower postprandial insulin and CPR values. Similar effects have been reported in studies examining the administration of nateglinide, a medication that restores early-phase insulin secretion [59].

In this study, after 6 days of HCHFD, adiponectin and FGF-21 levels were increased in non-obese subjects. Previous studies have also reported elevated plasma adiponectin concentrations after HCHFD for 3 days [23] and 7 days [24]. FGF-21 secretion is enhanced by HFD through peroxisome proliferator-activated receptor activation in the liver. Previous studies in rodents have demonstrated that adiponectin reduces levels of intracellular lipids in muscle and the liver by enhancing β-oxidation and maintaining normal insulin sensitivity [60,61,62,63]. In addition, FGF-21 has been shown to play a role in promoting the browning of white adipose tissue and increasing the thermogenic capacity of brown and beige adipose tissue [64]. On the other hand, FGF-21 inhibits lipolysis and promotes adiponectin secretion in white adipose tissue, thus reducing intracellular lipid accumulation and improving insulin sensitivity in muscle and the liver [25,26,27]. In the context of our study, the observed increases in adiponectin and FGF-21 levels following the HCHFD may have played a role in the metabolic changes we observed. For instance, the increase in adiponectin could have contributed to the maintenance of insulin sensitivity despite the high-fat diet, while the increase in FGF-21 might have helped mitigate the diet-induced increase in intracellular lipid accumulation. This suggests that these hormones may serve a compensatory role in response to a high-fat diet, helping to counteract fat accumulation in muscle and the liver.

The present study shows that fasting FFA levels decreased by 43%, total ketone body levels decreased by 53%, and glucose oxidation during sleep was consistently higher. In the previous study, sleep RQ decreased after HFD for 3 days [65]. However, in the present study, sleep RQ was higher and negatively corelated with fasting FFA levels (Supplementary Figure S1). Total ketone body levels were significantly lower after HCHFD. These results indicate that carbohydrate oxidation was enhanced after HCHFD in the present study, which was totally unexpected. Since a reduction in FFA levels due to HCHFD was also observed in a crossover study [11], it is reasonable to assume that HCHFD contributes to a decrease in FFA levels, at least partially, independently of study design. As previously discussed, higher FGF-21 concentrations might partly contribute to this phenomenon because injection of FGF-21 inhibited lipolysis and decreased plasma FFA levels in a few hours [25]. On the other hand, FFA release from adipose tissue is inhibited by insulin and promoted by catecholamines. Although the inhibition of FFA release by insulin is reduced by weight gain, a recent report has demonstrated that obesity and HFD cause downregulation of β3-adrenergic receptors in adipocytes through elevated inflammatory response, resulting in impaired FFA release from adipose tissue by catecholamines (catecholamine resistance) [66]. Thus, it is hypothesized that HCHFD causes catecholamine resistance, resulting in lower fasting FFA levels. Further studies are required to test this hypothesis.

Asians have a lower capacity to store fat in adipose tissue than Caucasians and fat easily accumulates in non-adipose tissues such as muscle and the liver [53,67]. Consequently, HCHFD might be expected to elevate FFA levels in Asians, potentially contributing to increased IMCL and IHL. However, in our study, we observed increases in IMCL and IHL despite a decrease in FFA levels, FFA being an important source of both IMCL and IHL. This finding suggests that dietary fatty acids, rather than elevated FFA levels, could be directly responsible for the increase in IMCL and IHL. Intriguingly, a previous report showed that after a short period of HCHFD, fasting FFA levels remained unchanged in both Caucasian and South Asian groups, while IHL increased similarly in both groups [10]. However, the potential ethnic differences in ectopic fat accumulation in the liver and skeletal muscle following short-term HCHFD remain unclear and warrant further investigation.

The present study has several limitations. First, we chose to focus on Japanese males in this study due to their higher prevalence of metabolic diseases compared to Japanese females, which consequently limits the generalizability of our results to females and other ethnic groups. Metabolism, fat distribution, and insulin sensitivity are known to vary between genders and among different ethnic groups. For example, differences in body composition, fat distribution, and hormonal regulation of metabolism between the sexes could potentially influence the effects of a HCHFD on insulin sensitivity and other metabolic parameters. In the present study, to avoid the potential confounding effects of sex, and given that the prevalence of metabolic diseases is much higher in Japanese men than Japanese women [68], we only included men. Furthermore, these factors also have been demonstrated to differ between East Asians and other ethnicities, including South Asians, Whites, African Caribbean Blacks, and Hispanics [14,15,16,69,70], suggesting that our findings may not be directly applicable to other populations. However, the fundamental mechanisms we studied could potentially operate in other populations, albeit with variations due to genetic, lifestyle, and environmental factors. Therefore, while our study provides valuable insights, they should be confirmed and expanded upon in diverse populations. Future studies should consider including both men and women and a variety of ethnic groups to provide a more comprehensive understanding of the metabolic effects of a HCHFD. Second, the number of subjects was relatively small. However, to precisely evaluate tissue-specific insulin sensitivity, we used the two-step clamp method, which is very complicated. Thus, we believe that 21 subjects do not represent a small sample. Third, another limitation is the lack of multiple comparison adjustment in secondary/exploratory outcomes. As our study is exploratory in nature, we did not initially apply these corrections; however, readers should interpret the results with caution due to the potential for inflated Type I error rates. Finally, the associations shown in the present study do not indicate causality. Further study is required to confirm causal relationships among metabolic parameters.

In conclusion, short-term HCHFD caused ectopic fat accumulation and impaired insulin sensitivity in liver and muscle. However, higher serum insulin concentrations completely compensated for impaired insulin sensitivity via decreased insulin clearance and increased endogenous insulin secretion during hyperinsulinemia.

## Figures and Tables

**Table 1:** Anthropometric and metabolic characteristics and blood sample results of 21 healthy non-obese men at baseline and after short-term HCHFD.

	Baseline	After HCHFD	*p* Value
Body weight (kg)	61.6 ± 6.1	62.0 ± 6.1	**0.021**
Fat free mass (kg)	52.8 ± 5.3	53.4 ± 5.4	**0.015**
Body mass index (kg/m^2^)	20.6 ± 1.8	20.7 ± 1.7	**0.019**
Fat mass (kg)	6.7 ± 1.4	6.6 ± 1.5	0.727
Percent body fat (%)	13.1 ± 3.1	12.7 ± 3.1	0.116
Serum insulin (μIU/mL)	4.7 ± 1.2	5.5 ± 2.4	0.274
C-peptide (ng/mL)	1.1 ± 0.2	1.2 ± 0.3	0.099
Plasma glucose (mg/dL)	86.6 ± 5.6	90.5 ± 5.4	**0.012**
Free fatty acids (μEq/L)	665.0 ± 229.4	377.3 ± 180.6	**<0.001**
Total cholesterol (mg/dL)	152.8 ± 19.7	161.0 ± 23.2	**0.022**
High-density lipoprotein cholesterol (mg/dL)	52.6 ± 11.0	59.3 ± 11.0	**0.001**
Low-density lipoprotein cholesterol (mg/dL)	88.2 ± 19.6	92.0 ± 21.6	0.163
Triglycerides (mg/dL)	66.5 ± 14.8	70.5 ± 28.4	0.412
Aspartate aminotransferase (U/L)	18.7 ± 5.2	18.4 ± 5.2	0.689
Alanine aminotransferase (U/L)	15.6 ± 6.5	17.4 ± 6.9	0.095
Alkaline phosphatase (U/L)	189.5 ± 44.4	203.6 ± 47.0	0.073
γ-Glutamyl transpeptidase (U/L)	15.9 ± 3.9	14.6 ± 3.2	**0.007**
Total bile acid (μmol/L)	5.4 ± 5.0	7.7 ± 8.5	**0.040**
Acetoacetic acid (μmol/L)	29.5 ± 23.3	19.1 ± 11.5	**0.021**
3-hydroxyacetic acid (μmol/L)	82.6 ± 72.4	33.2 ± 23.4	**<0.001**
Total ketone bodies (μmol/L)	112.1 ± 95.3	52.3 ± 34.2	**0.002**
Total adiponectin (μg/mL)	5.37 ± 2.15	5.80 ± 2.20	**0.004**
High-molecular-weight adiponectin (μg/mL)	1.93 ± 1.30	2.14 ± 1.42	**0.021**
FGF-21 (pg/mL)	107.5 ± 60.0	169.7 ± 107.4	**0.001**
Interleukin 6 (pg/mL)	1.2 ± 2.3	1.2 ± 1.7	0.984
Leptin (ng/mL)	4.5 ± 1.6	5.0 ± 1.7	**0.048**
Monocyte chemoattractant protein-1 (pg/mL)	200.1 ± 46.7	200.3 ± 32.8	0.768
LBP (μg/mL)	4.9 ± 0.7	5.6 ± 0.7	**0.006**
High-sensitivity CRP (ng/mL)	157.0 ± 308.8	224.3 ± 228.6	0.073
Tumor necrosis factor alpha (pg/mL)	0.70 ± 0.18	0.68 ± 0.16	0.365
Basal metabolic rate (kcal/min)	0.94 ± 0.12	0.95 ± 0.12	0.509
Basal RQ	0.89 ± 0.08	0.92 ± 0.05	0.103
Sleep energy expenditure (kcal/min)	0.87 ± 0.09	0.88 ± 0.09	0.407
Sleep RQ	0.83 ± 0.03	0.87 ± 0.04	**<0.001**

Data are means ± SD. Bold text represents statistical significance (*p* < 0.05). Abbreviations: FGF-21: fibroblast growth factor 21; LBP: lipopolysaccharide binding protein; CRP: C-reactive protein; RQ: respiratory quotient.

**Table 2:** Fat distribution and two-step hyperinsulinemic euglycemic clamp data at baseline and after short-term high-calorie high-fat diet (HCHFD).

	Baseline	After HCHFD	*p* Value
Intrahepatic lipid (%)	0.5 ± 0.4	1.5 ± 1.0	**<0.001**
Intramyocellular lipid-TA (S-fat/Cre)	1.7 ± 1.2	2.5 ± 1.3	**0.009**
Intramyocellular lipid-SOL (S-fat/Cre)	5.2 ± 2.7	6.8 ± 2.7	**0.005**
Abdominal visceral fat area (cm^2^)	46.0 ± 11.2	46.6 ± 10.2	0.476
Abdominal subcutaneous fat area (cm^2^)	71.7 ± 26.4	73.7 ± 26.8	0.217
SS_SI_ during the first step (μU/mL)	17.1 ± 2.1	18.4 ± 2.7	**0.019**
SSsc during the first step (ng/mL)	0.72 ± 0.25	0.82 ± 0.32	**0.019**
SS_SI_ during the second step (μU/mL)	31.5 ± 4.1	34.4 ± 4.9	**0.002**
SSsc during the second step (ng/mL)	0.60 ± 0.27	0.73 ± 0.28	**0.006**
MCRI during the second step (mL/min per m^2^)	692.4 ± 94.1	653.1 ± 99.7	**0.004**
Basal EGP (mg/m^2^/min)	76.9 ± 3.4	79.4 ± 4.7	**0.023**
% reduction in EGP during the first step (%/μU)	75.2 ± 11.6	73.5 ± 13.6	0.419
% reduction in EGP/SS_SI_ during the first step (%/μU·mL^−1^)	4.46 ± 0.85	4.09 ± 0.97	**0.044**
Rd during the second step (mg/kg FFM/min^−1^)	7.49 ± 1.20	7.67 ± 1.29	0.268
Rd/SS_SI_ during the second step (mg/kg FFM·min^−1^/μU·mL^−1^)	0.24 ± 0.06	0.23 ± 0.05	**0.042**
Adipose tissue insulin resistance index	3209.5 ± 1519.6	2345.1 ± 2012.5	**0.023**

Data are means ± SD. Bold text represents statistical significance (*p* < 0.05). Abbreviations: SOL: soleus; TA: tibialis anterior; S-fat: methylene signal intensity; Cre: creatine signal intensity; SS_SI_: steady-state serum insulin; SSsc: steady-state serum C-peptide; MCRI: metabolic clearance rate of insulin; EGP: endogenous glucose production; Rd: rate of glucose disappearance; FFM: fat-free mass; IR: insulin resistance.

**Table 3:** Meal load test data at baseline and after short-term high-calorie high-fat diet (HCHFD).

	Baseline	After HCHFD	*p* Value
AUC-glucose (mg/dL × min × 10^3^)	26.9 ± 27.0	25.9 ± 2.7	0.131
AUC-FFA (μEq/L × min × 10^3^)	41.6 ± 8.2	32.2 ± 6.3	**<0.001**
AUC-insulin (IU/mL × min × 10^3^)	9.0 ± 3.8	7.7 ± 2.7	**0.039**
AUC-CPR (ng/mL × min × 10^3^)	1.3 ± 0.3	1.1 ± 0.3	**0.007**
AUC-glucagon (pg/mL × min × 10^3^)	7.4 ± 2.8	8.3 ± 3.1	0.140
AUC-GIP (pmol/L × min × 10^3^)	10.5 ± 2.8	10.5 ± 3.0	0.986
AUC-GLP-1 (pmol/L × min × 10^3^)	0.85 ± 0.38	0.90 ± 0.40	0.339
Insulin sensitivity index (comp)	10.0 ± 5.5	9.5 ± 4.6	0.794
Insulinogenic index	0.9 ± 0.3	1.3 ± 0.6	**<0.001**
Insulinogenic index (c-peptide)	0.072 ± 0.022	0.101 ± 0.049	**<0.001**

Data are means ± SD. Bold text represents statistical significance *(p <* 0.05). Abbreviations: AUC: area under the curve; FFA: free fatty acid; CPR: c-peptide; GIP: gastric inhibitory polypeptide; GLP-1: glucagon-like peptide; ISI: insulin sensitivity index.

**Table 4:** Abundance of gut microbiota at the phylum level at baseline and after short-term high-calorie high-fat diet (HCHFD).

	Baseline	After HCHFD	*p* Value
*Actinobacteria* (%)	6.89 ± 4.86	9.73 ± 5.66	**0.005**
*Bacteroidetes* (%)	25.19 ± 7.88	25.79 ± 9.00	0.970
*Firmicutes* (%)	63.05 ± 9.72	60.91 ± 11.14	0.232
*Fusobacteria* (%)	1.75 ± 2.76	1.41 ± 2.59	0.156
*Proteobacteria* (%)	2.82 ± 3.33	2.16 ± 1.04	0.970

Data are means ± SD. Bold text represents statistical significance (*p* < 0.05).

## Data Availability

The datasets generated during and/or analyzed during the current study are available from the corresponding author upon reasonable request. Yoshifumi Tamura is the guarantor of this work and, as such, had full access to all the data in the study and takes responsibility for the integrity of the data and the accuracy of the data analysis.

## Supplementary Materials

The following supporting information can be downloaded at: https://www.mdpi.com/article/10.3390/jcm12124084/s1, Figure S1: Changes in individual fasting glucose, insulin, and C-peptide during the meal test before and after the HCHFD. The red line represents the average. Figure S2: Changes in the a-diversity and b-diversity before and after the HCHFD. a-diversity, which includes observed species, Chao1, and Shannon phylogenetic diversity indices, was assessed and analyzed utilizing the Wilcoxon rank sum test. b-diversity was approximated through the application of the weighted UniFrac metric. Figure S3: Correlation between percent change in sleep RQ and percent change in fasting FFA.

## References

[1] Chan J.C., Malik V., Jia W., Kadowaki T., Yajnik C.S., Yoon K.H., Hu F.B. (2009). Diabetes in Asia: Epidemiology, risk factors, and pathophysiology. JAMA.

[2] Reaven G.M. (1988). Banting lecture 1988. Role of insulin resistance in human disease. Diabetes.

[3] Katsuki A., Sumida Y., Urakawa H., Gabazza E.C., Murashima S., Maruyama N., Morioka K., Nakatani K., Yano Y., Adachi Y. (2003). Increased visceral fat and serum levels of triglyceride are associated with insulin resistance in Japanese metabolically obese, normal weight subjects with normal glucose tolerance. Diabetes Care.

[4] Takeno K., Tamura Y., Kawaguchi M., Kakehi S., Watanabe T., Funayama T., Furukawa Y., Kaga H., Yamamoto R., Kim M. (2016). Relation between Insulin Sensitivity and Metabolic Abnormalities in Japanese Men with BMI of 23–25 kg/m^2^. J. Clin. Endocrinol. Metab..

[5] Chooi Y.C., Ding C., Chan Z., Choo J., Sadananthan S.A., Michael N., Lee Y., Velan S.S., Magkos F. (2018). Moderate Weight Loss Improves Body Composition and Metabolic Function in Metabolically Unhealthy Lean Subjects. Obesity.

[6] Ding C., Chan Z., Chooi Y.C., Choo J., Sadananthan S.A., Chang A., Sasikala S., Michael N., Velan S.S., Magkos F. (2018). Regulation of glucose metabolism in nondiabetic, metabolically obese normal-weight Asians. Am. J. Physiol.-Endocrinol. Metab..

[7] Micha R., Mozaffarian D. (2010). Saturated fat and cardiometabolic risk factors, coronary heart disease, stroke, and diabetes: A fresh look at the evidence. Lipids.

[8] de Souza R.J., Mente A., Maroleanu A., Cozma A.I., Ha V., Kishibe T., Uleryk E., Budylowski P., Schünemann H., Beyene J. (2015). Intake of saturated and trans unsaturated fatty acids and risk of all cause mortality, cardiovascular disease, and type 2 diabetes: Systematic review and meta-analysis of observational studies. BMJ.

[9] Funaki M. (2009). Saturated fatty acids and insulin resistance. J. Med. Investig..

[10] Bakker L.E., van Schinkel L.D., Guigas B., Streefland T.C., Jonker J.T., van Klinken J.B., van der Zon G.C., Lamb H.J., Smit J.W., Pijl H. (2014). A 5-day high-fat, high-calorie diet impairs insulin sensitivity in healthy, young South Asian men but not in Caucasian men. Diabetes.

[11] Brons C., Jensen C.B., Storgaard H., Hiscock N.J., White A., Appel J.S., Jacobsen S., Nilsson E., Larsen C.M., Astrup A. (2009). Impact of short-term high-fat feeding on glucose and insulin metabolism in young healthy men. J. Physiol..

[12] Ott B., Skurk T., Lagkouvardos L., Fischer S., Buttner J., Lichtenegger M., Clavel T., Lechner A., Rychlik M., Haller D. (2018). Short-Term Overfeeding with Dairy Cream Does Not Modify Gut Permeability, the Fecal Microbiota, or Glucose Metabolism in Young Healthy Men. J. Nutr..

[13] Adochio R.L., Leitner J.W., Gray K., Draznin B., Cornier M.A. (2009). Early responses of insulin signaling to high-carbohydrate and high-fat overfeeding. Nutr. Metab..

[14] Tai E.S., Lim S.C., Chew S.K., Tan B.Y., Tan C.E. (2000). Homeostasis model assessment in a population with mixed ethnicity: The 1992 Singapore National Health Survey. Diabetes Res. Clin. Pract..

[15] Khoo C.M., Leow M.K., Sadananthan S.A., Lim R., Venkataraman K., Khoo E.Y., Velan S.S., Ong Y.T., Kambadur R., McFarlane C. (2014). Body fat partitioning does not explain the interethnic variation in insulin sensitivity among Asian ethnicity: The Singapore adults metabolism study. Diabetes.

[16] Kanaya A.M., Herrington D., Vittinghoff E., Ewing S.K., Liu K., Blaha M.J., Dave S.S., Qureshi F., Kandula N.R. (2014). Understanding the high prevalence of diabetes in U.S. south Asians compared with four racial/ethnic groups: The MASALA and MESA studies. Diabetes Care.

[17] Ling C., Ronn T. (2019). Epigenetics in Human Obesity and Type 2 Diabetes. Cell Metab..

[18] Saad M.J., Santos A., Prada P.O. (2016). Linking Gut Microbiota and Inflammation to Obesity and Insulin Resistance. Physiology.

[19] Kawano Y., Nakae J., Watanabe N., Kikuchi T., Tateya S., Tamori Y., Kaneko M., Abe T., Onodera M., Itoh H. (2016). Colonic Pro-inflammatory Macrophages Cause Insulin Resistance in an Intestinal Ccl2/Ccr2-Dependent Manner. Cell Metab..

[20] André P., Laugerette F., Féart C. (2019). Metabolic Endotoxemia: A Potential Underlying Mechanism of the Relationship between Dietary Fat Intake and Risk for Cognitive Impairments in Humans?. Nutrients.

[21] Kakehi S., Tamura Y., Takeno K., Sakurai Y., Kawaguchi M., Watanabe T., Funayama T., Sato F., Ikeda S., Kanazawa A. (2016). Increased intramyocellular lipid/impaired insulin sensitivity is associated with altered lipid metabolic genes in muscle of high responders to a high-fat diet. Am. J. Physiol.-Endocrinol. Metab..

[22] Kawaguchi M., Tamura Y., Kakehi S., Takeno K., Sakurai Y., Watanabe T., Funayama T., Sato F., Ikeda S., Ogura Y. (2014). Association Between Expression of FABPpm in Skeletal Muscle and Insulin Sensitivity in Intramyocellular Lipid-Accumulated Nonobese Men. J. Clin. Endocrinol. Metab..

[23] Heilbronn L.K., Campbell L.V., Xu A., Samocha-Bonet D. (2013). Metabolically protective cytokines adiponectin and fibroblast growth factor-21 are increased by acute overfeeding in healthy humans. PLoS ONE.

[24] Cahill F., Amini P., Wadden D., Khalili S., Randell E., Vasdev S., Gulliver W., Sun G. (2013). Short-term overfeeding increases circulating adiponectin independent of obesity status. PLoS ONE.

[25] Li X., Ge H., Weiszmann J., Hecht R., Li Y.S., Véniant M.M., Xu J., Wu X., Lindberg R., Li Y. (2009). Inhibition of lipolysis may contribute to the acute regulation of plasma FFA and glucose by FGF21 in *ob*/*ob* mice. FEBS Lett..

[26] She Q.Y., Bao J.F., Wang H.Z., Liang H., Huang W., Wu J., Zhong Y., Ling H., Li A., Qin S.L. (2022). Fibroblast growth factor 21: A “rheostat” for metabolic regulation?. Metabolism.

[27] Szczepanska E., Gietka-Czernel M. (2022). FGF21: A Novel Regulator of Glucose and Lipid Metabolism and Whole-Body Energy Balance. Horm. Metab. Res..

[28] McNamara E.A., Kilim H.P., Malabanan A.O., Whittaker L.G., Rosen H.N. (2018). Enhanced Precision of the New Hologic Horizon Model Compared With the Old Discovery Model Is Less Evident When Fewer Vertebrae Are Included in the Analysis. J. Clin. Densitom..

[29] Tamura Y., Tanaka Y., Sato F., Choi J.B., Watada H., Niwa M., Kinoshita J., Ooka A., Kumashiro N., Igarashi Y. (2005). Effects of diet and exercise on muscle and liver intracellular lipid contents and insulin sensitivity in type 2 diabetic patients. J. Clin. Endocrinol. Metab..

[30] Sato F., Tamura Y., Watada H., Kumashiro N., Igarashi Y., Uchino H., Maehara T., Kyogoku S., Sunayama S., Sato H. (2007). Effects of diet-induced moderate weight reduction on intrahepatic and intramyocellular triglycerides and glucose metabolism in obese subjects. J. Clin. Endocrinol. Metab..

[31] Kelley D.E., McKolanis T.M., Hegazi R.A., Kuller L.H., Kalhan S.C. (2003). Fatty liver in type 2 diabetes mellitus: Relation to regional adiposity, fatty acids, and insulin resistance. Am. J. Physiol.-Endocrinol. Metab..

[32] Kotronen A., Seppälä-Lindroos A., Bergholm R., Yki-Järvinen H. (2008). Tissue specificity of insulin resistance in humans: Fat in the liver rather than muscle is associated with features of the metabolic syndrome. Diabetologia.

[33] Basu R., Barosa C., Jones J., Dube S., Carter R., Basu A., Rizza R.A. (2013). Pathogenesis of prediabetes: Role of the liver in isolated fasting hyperglycemia and combined fasting and postprandial hyperglycemia. J. Clin. Endocrinol. Metab..

[34] Lindegaard B., Frosig C., Petersen A.M., Plomgaard P., Ditlevsen S., Mittendorfer B., Van Hall G., Wojtaszewski J.F., Pedersen B.K. (2007). Inhibition of lipolysis stimulates peripheral glucose uptake but has no effect on endogenous glucose production in HIV lipodystrophy. Diabetes.

[35] Maggs D.G., Buchanan T.A., Burant C.F., Cline G., Gumbiner B., Hsueh W.A., Inzucchi S., Kelley D., Nolan J., Olefsky J.M. (1998). Metabolic effects of troglitazone monotherapy in type 2 diabetes mellitus. A randomized, double-blind, placebo-controlled trial. Ann. Intern. Med..

[36] Watanabe T., Tamura Y., Kakehi S., Funayama T., Gastaldelli A., Takeno K., Kawaguchi M., Yamamoto R., Sato F., Ikeda S. (2015). Effects of sitagliptin on ectopic fat contents and glucose metabolism in type 2 diabetic patients with fatty liver: A pilot study. J. Diabetes Investig..

[37] Steele R. (1959). Influences of glucose loading and of injected insulin on hepatic glucose output. Ann. N. Y. Acad. Sci..

[38] Groop L.C., Bonadonna R.C., DelPrato S., Ratheiser K., Zyck K., Ferrannini E., DeFronzo R.A. (1989). Glucose and free fatty acid metabolism in non-insulin-dependent diabetes mellitus. Evidence for multiple sites of insulin resistance. J. Clin. Investig..

[39] Kotronen A., Juurinen L., Tiikkainen M., Vehkavaara S., Yki-Järvinen H. (2008). Increased liver fat, impaired insulin clearance, and hepatic and adipose tissue insulin resistance in type 2 diabetes. Gastroenterology.

[40] Abdul-Ghani M., DeFronzo R.A. (2007). Fasting hyperglycemia impairs glucose- but not insulin-mediated suppression of glucagon secretion. J. Clin. Endocrinol. Metab..

[41] Kaga H., Tamura Y., Takeno K., Kakehi S., Funayama T., Furukawa Y., Nishitani-Yokoyama M., Shimada K., Daida H., Aoki S. (2017). Correlates of insulin clearance in apparently healthy non-obese Japanese men. Sci. Rep..

[42] Takagi T., Naito Y., Inoue R., Kashiwagi S., Uchiyama K., Mizushima K., Tsuchiya S., Dohi O., Yoshida N., Kamada K. (2019). Differences in gut microbiota associated with age, sex, and stool consistency in healthy Japanese subjects. J. Gastroenterol..

[43] Uchida F., Oh S., Shida T., Suzuki H., Yamagata K., Mizokami Y., Bukawa H., Tanaka K., Shoda J. (2021). Effects of Exercise on the Oral Microbiota and Saliva of Patients with Non-Alcoholic Fatty Liver Disease. Int. J. Environ. Res. Public Health.

[44] Katayose Y., Tasaki M., Ogata H., Nakata Y., Tokuyama K., Satoh M. (2009). Metabolic rate and fuel utilization during sleep assessed by whole-body indirect calorimetry. Metabolism.

[45] Tokuyama K., Ogata H., Katayose Y., Satoh M. (2009). Algorithm for transient response of whole body indirect calorimeter: Deconvolution with a regularization parameter. J. Appl. Physiol..

[46] Kobayashi F., Ogata H., Omi N., Nagasaka S., Yamaguchi S., Hibi M., Tokuyama K. (2014). Effect of breakfast skipping on diurnal variation of energy metabolism and blood glucose. Obes. Res. Clin. Pract..

[47] Sato M., Nakamura K., Ogata H., Miyashita A., Nagasaka S., Omi N., Yamaguchi S., Hibi M., Umeda T., Nakaji S. (2011). Acute effect of late evening meal on diurnal variation of blood glucose and energy metabolism. Obes. Res. Clin. Pract..

[48] Ferrannini E. (1988). The theoretical bases of indirect calorimetry: A review. Metabolism.

[49] Yajima K., Iwayama K., Ogata H., Park I., Tokuyama K. (2018). Meal rich in rapeseed oil increases 24-h fat oxidation more than meal rich in palm oil. PLoS ONE.

[50] Matsuda M., DeFronzo R.A. (1999). Insulin sensitivity indices obtained from oral glucose tolerance testing: Comparison with the euglycemic insulin clamp. Diabetes Care.

[51] Rijkelijkhuizen J.M., Girman C.J., Mari A., Alssema M., Rhodes T., Nijpels G., Kostense P.J., Stein P.P., Eekhoff E.M., Heine R.J. (2009). Classical and model-based estimates of beta-cell function during a mixed meal vs. an OGTT in a population-based cohort. Diabetes Res. Clin. Pract..

[52] Brodovicz K.G., Girman C.J., Simonis-Bik A.M., Rijkelijkhuizen J.M., Zelis M., Bunck M.C., Mari A., Nijpels G., Eekhoff E.M., Dekker J.M. (2011). Postprandial metabolic responses to mixed versus liquid meal tests in healthy men and men with type 2 diabetes. Diabetes Res. Clin. Pract..

[53] Sugimoto D., Tamura Y., Takeno K., Kaga H., Someya Y., Kakehi S., Funayama T., Furukawa Y., Suzuki R., Kadowaki S. (2019). Clinical Features of Nonobese, Apparently Healthy, Japanese Men With Reduced Adipose Tissue Insulin Sensitivity. J. Clin. Endocrinol. Metab..

[54] Moreira A.P.B., Texeira T.F.S., Ferreira A.B., Peluzio M.D.C.G., Alfenas R.D.C.G. (2012). Influence of a high-fat diet on gut microbiota, intestinal permeability and metabolic endotoxaemia. Br. J. Nutr..

[55] Zhang Y.J., Li S., Gan R.Y., Zhou T., Xu D.P., Li H.B. (2015). Impacts of gut bacteria on human health and diseases. Int. J. Mol. Sci..

[56] Kaga H., Tamura Y., Takeno K., Kakehi S., Someya Y., Funayama T., Furukawa Y., Suzuki R., Sugimoto D., Kadowaki S. (2019). Higher C-Peptide Level During Glucose Clamp Is Associated With Muscle Insulin Resistance in Nonobese Japanese Men. J. Endocr. Soc..

[57] Foley K.P., Zlitni S., Duggan B.M., Barra N.G., Anhê F.F., Cavallari J.F., Henriksbo B.D., Chen C.Y., Huang M., Lau T.C. (2020). Gut microbiota impairs insulin clearance in obese mice. Mol. Metab..

[58] Huang J., Ledford K.J., Pitkin W.B., Russo L., Najjar S.M., Siragy H.M. (2013). Targeted deletion of murine CEACAM 1 activates PI3K-Akt signaling and contributes to the expression of (Pro)renin receptor via CREB family and NF-κB transcription factors. Hypertension.

[59] Uchino H., Niwa M., Shimizu T., Nishiyama K., Kawamori R. (2000). Impairment of early insulin response after glucose load, rather than insulin resistance, is responsible for postprandial hyperglycemia seen in obese type 2 diabetes: Assessment using nateglinide, a new insulin secretagogue. Endocr. J..

[60] Yamauchi T., Kadowaki T. (2008). Physiological and pathophysiological roles of adiponectin and adiponectin receptors in the integrated regulation of metabolic and cardiovascular diseases. Int. J. Obes..

[61] Kadowaki T., Yamauchi T., Kubota N., Hara K., Ueki K., Tobe K. (2006). Adiponectin and adiponectin receptors in insulin resistance, diabetes, and the metabolic syndrome. J. Clin. Investig..

[62] Yamauchi T., Kamon J., Waki H., Terauchi Y., Kubota N., Hara K., Mori Y., Ide T., Murakami K., Tsuboyama-Kasaoka N. (2001). The fat-derived hormone adiponectin reverses insulin resistance associated with both lipoatrophy and obesity. Nat. Med..

[63] Yamauchi T., Kamon J., Minokoshi Y., Ito Y., Waki H., Uchida S., Yamashita S., Noda M., Kita S., Ueki K. (2002). Adiponectin stimulates glucose utilization and fatty-acid oxidation by activating AMP-activated protein kinase. Nat. Med..

[64] Flippo K.H., Potthoff M.J. (2021). Metabolic Messengers: FGF21. Nat. Metab..

[65] Ukropcova B., Sereda O., de Jonge L., Bogacka I., Nguyen T., Xie H., Bray G.A., Smith S.R. (2007). Family history of diabetes links impaired substrate switching and reduced mitochondrial content in skeletal muscle. Diabetes.

[66] Valentine J.M., Ahmadian M., Keinan O., Abu-Odeh M., Zhao P., Zhou X., Keller M.P., Gao H., Yu R.T., Liddle C. (2022). β3-Adrenergic receptor downregulation leads to adipocyte catecholamine resistance in obesity. J. Clin. Investig..

[67] Rattarasarn C. (2018). Dysregulated lipid storage and its relationship with insulin resistance and cardiovascular risk factors in non-obese Asian patients with type 2 diabetes. Adipocyte.

[68] Arai H., Yamamoto A., Matsuzawa Y., Saito Y., Yamada N., Oikawa S., Mabuchi H., Teramoto T., Sasaki J., Nakaya N. (2006). Prevalence of metabolic syndrome in the general Japanese population in 2000. J. Atheroscler. Thromb..

[69] Nazare J.A., Smith J.D., Borel A.L., Haffner S.M., Balkau B., Ross R., Massien C., Alméras N., Després J.P. (2012). Ethnic influences on the relations between abdominal subcutaneous and visceral adiposity, liver fat, and cardiometabolic risk profile: The International Study of Prediction of Intra-Abdominal Adiposity and Its Relationship With Cardiometabolic Risk/Intra-Abdominal Adiposity. Am. J. Clin. Nutr..

[70] Azuma K., Kadowaki T., Cetinel C., Kadota A., El-Saed A., Kadowaki S., Edmundowicz D., Nishio Y., Sutton-Tyrrell K., Okamura T. (2009). Higher liver fat content among Japanese in Japan compared with non-Hispanic whites in the United States. Metabolism.

