# Supplementary material for: A Short-Term High-Fat Diet Worsens Insulin Sensitivity with Changes in Metabolic Parameters in Non-Obese Japanese Men
Source: J Clin Med. 2023 Jun 16;12(12):4084. doi: 10.3390/jcm12124084 (PMC10299415; doi:10.3390/jcm12124084)
Supplement: Supplementary file 1 [file jcm-12-04084-s001.zip › jcm-2435363-supplementary.pdf]

# Supplementary Figure S1

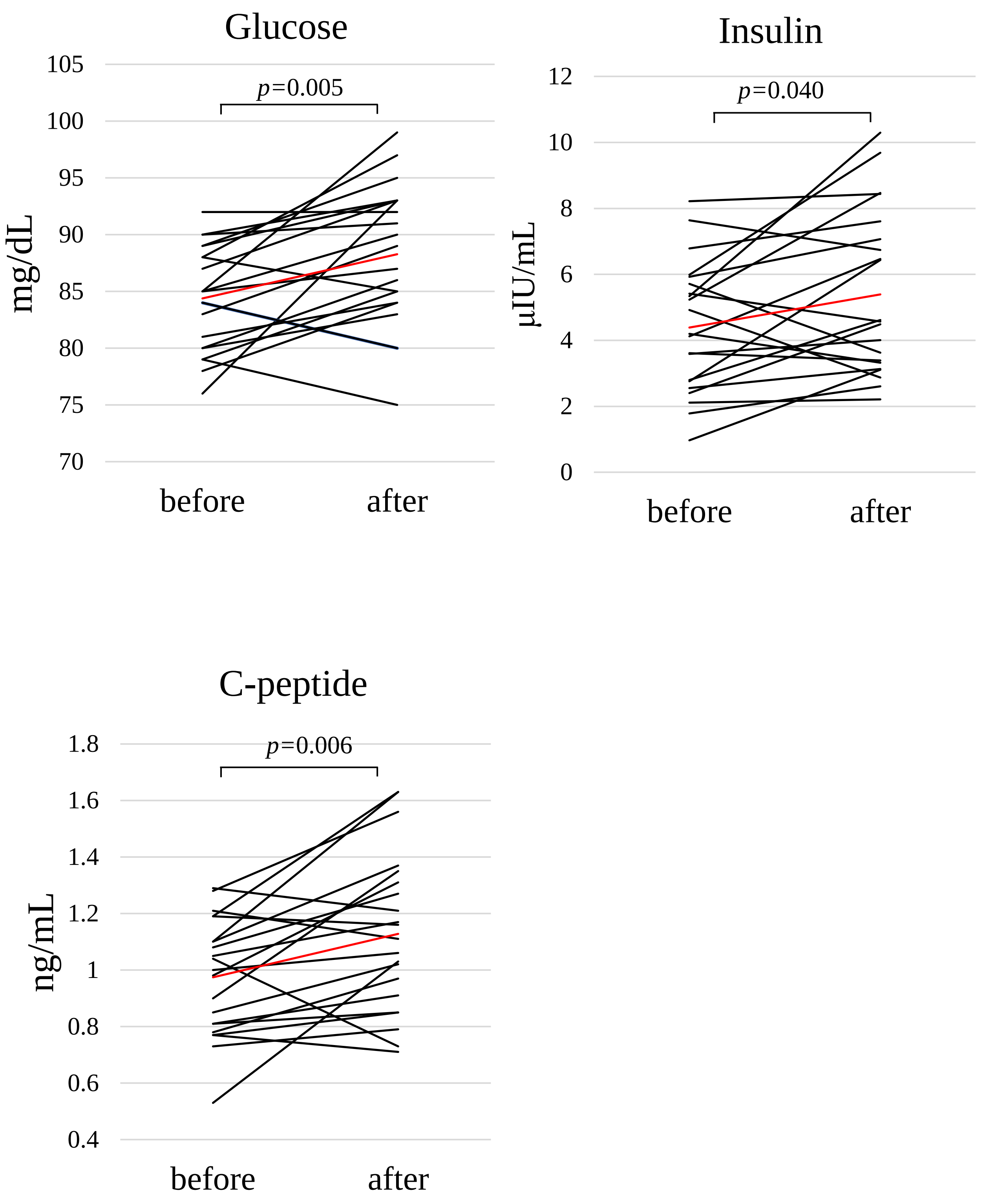


Changes in individual fasting glucose, insulin, and C-peptide during the meal test before and after the HCHFD. The red line represents the average.

Supplementary Figure S2

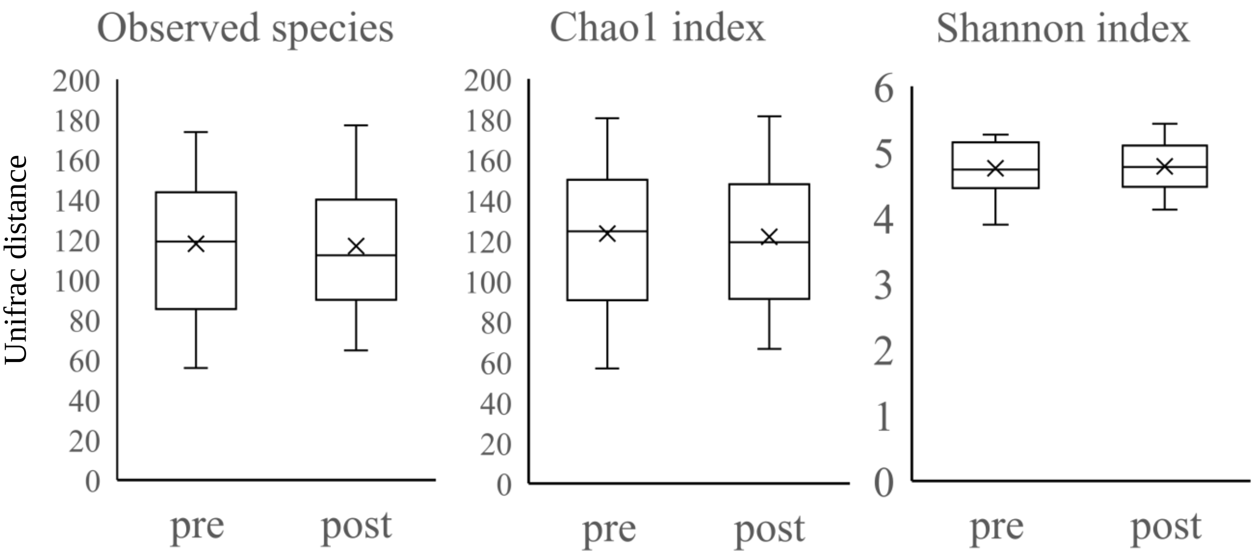


α-diversity

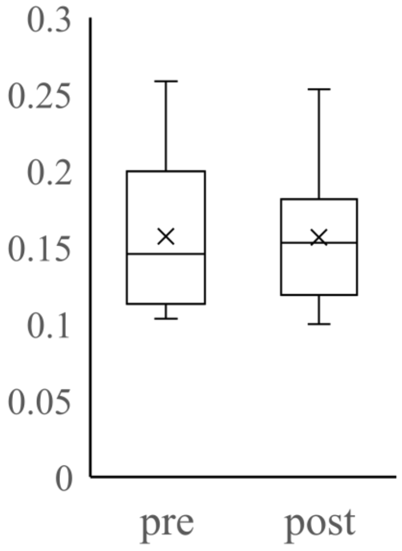


β-diversity

Changes in the α-diversity and β-diversity before and after the HCHFD. α-diversity, which includes observed species, Chao1, and Shannon phylogenetic diversity indices, was assessed and analyzed utilizing the Wilcoxon rank sum test . β-diversity was approximated through the application of the weighted UniFrac metric.

# Supplementary Figure S3

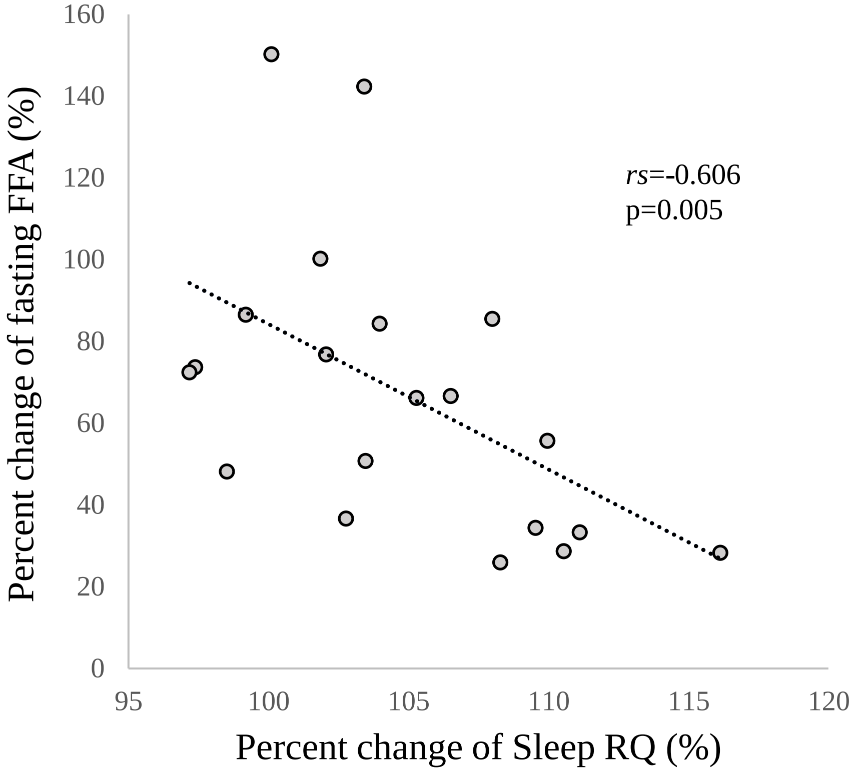


Correlation between percent change in sleep RQ and percent change in fasting FFA.
